# The impact of family communication patterns on filial responsibilities among college students: a chain mediation model of perceived social support and gratitude

**DOI:** 10.3389/fpsyg.2026.1708482

**Published:** 2026-04-02

**Authors:** Jiebing Ma, Yizhan Du, Chengchieh Li, Guoyao Lin

**Affiliations:** 1 Department of Psychology, Minnan Normal University, Zhangzhou, China; 2 Department of Applied Psychology, Fujian Polytechnic Normal University, Fuzhou, China; 3 Department of Psychology, Shaoguan University, Shaoguan, China; 4 Department of Psychology, Asia University, Taichung, Taiwan

**Keywords:** conformity orientation, conversation orientation, family communication patterns, filial responsibility, gratitude, perceived social support

## Abstract

To examine the impact of family communication patterns on college students’ filial responsibilities and the mechanisms underlying this relationship, a survey was administered to 800 students at a university in Fujian Province, employing the Family Communication Patterns Scale, Perceived Social Support Scale, Gratitude Scale, and Filial Responsibility Scale. The findings indicated the following: (1) The conversation orientation of family communication patterns (the degree of conversation orientation, which influences the extent to which family members can comfortably and freely discuss a wide range of topics) significantly and positively predicts college students’ filial responsibility, whereas the conformity orientation (the degree of conformity orientation, which determines how closely family members adhere to the family’s homogeneous beliefs, values, and hierarchical structure) significantly and negatively predicts it; (2) Perceived social support partially mediates the relationship between the conformity orientation of family communication patterns and students’ filial responsibility; (3) Gratitude also partially mediates the relationship between both the conversation and conformity orientations of family communication patterns and filial responsibility; (4) Moreover, perceived social support and gratitude together exert a chain-mediating effect between the conversation and conformity orientations of family communication patterns and college students’ filial responsibility. In conclusion, both the conversation and conformity orientations of family communication patterns directly influenced college students’ filial responsibilities, as well as exerted an indirect influence through perceived social support and gratitude.

## Introduction

In the context of an increasingly aging population, evolving family structures, and escalating living pressures in modern Chinese society, the harmony and stability of society depend on individuals fulfilling their filial duties and responsibilities toward their aging parents. This concept, referred to as “filial responsibility,” is defined as a social norm and cultural expectation that governs the care adult children provide to their elderly parents (Aires et al., 2019). It can be assessed through the attitudes and behaviors of adult children toward caregiving and supporting their parents (Chappell and Funk, 2011). Previous studies have indicated that the interaction process between children and family members (Cicirelli, 1989; Datta et al., 2005; Sung, 1995; Gans et al., 2009; Mikane et al., 2011; Kim et al., 2014; Hwang et al., 2018), perceived levels of social support (Zhang et al., 2018; Ron, 2016, 2019; Wang, 2023), and gratitude (Keller, 2006; Peng and Dai, 2019; Long et al., 2014; Wang et al., 2020) all influence their filial responsibility. College students, who transition from adolescence to early adulthood, must gradually detach from parental care and support while learning to independently manage their lives and assume greater social and familial responsibilities. Therefore, investigating the impact and development of their interactions with family on the filial responsibilities they are expected to assume, as well as the role of perceived social support and gratitude in this process, may attract the attention of scholars and parents in China.

Family communication patterns emerge through the interactive processes between family members. These patterns not only reflect the nature of interpersonal interactions within the family but also significantly influence these interactions (Horstman et al., 2018; Scruggs and Schrodt, 2021). Within the framework of family systems theory, family communication patterns highlight the reciprocal relationships among family members, a dynamic that profoundly shapes children’s attitudes, emotional experiences, and behavioral tendencies. Previous research has shown that children who maintain strong emotional bonds and high-quality relationships with their parents tend to exhibit an enhanced sense of filial responsibility (Gans et al., 2009; Kim et al., 2014; Hwang et al., 2018; Swartz, 2009; Lin et al., 2023). Liu et al. (2018) suggest that children perceive their filial responsibility through the positive emotional experiences derived from communication with their parents. Family communication patterns consist of two key interactive dimensions: (1) the degree of conversation orientation, which influences the extent to which family members can comfortably and freely discuss a wide range of topics (Bevan et al., 2019). This dimension facilitates the expression or concealment of personal thoughts and feelings, contributing to the development of a shared social reality (Schrodt, 2020), and (2) the degree of conformity orientation, which determines how closely family members adhere to the family’s homogeneous beliefs, values, and hierarchical structure (Bevan et al., 2019). This dimension influences their conformity or deviation from family values, beliefs, and norms (Schrodt, 2020). Based on these considerations, Hypothesis 1 is proposed: family communication patterns have a certain impact on the filial responsibility sense of college students.

The formation and development of filial responsibility are influenced not only by family communication patterns but also by the level of social support perceived by individuals. Previous research indicates that filial responsibility can be maintained through social support (Zhang et al., 2018). Furthermore, studies have shown that when children receive greater family support, their attitudes toward filial responsibility tend to be more positive and proactive (Ron, 2016, 2019; Wang, 2023). Both family systems theory and self-determination theory provide insights into how family communication patterns, mediated by perceived social support, predict the formation and development of filial responsibility. Family systems theory posits that family communication patterns influence parent-child interactions, which, in turn, extend to interpersonal relationships outside the family (Koesten, 2004; Ledbetter, 2009), thereby fostering the perception of social support (Luo et al., 2017; Liu and Xia, 2018; Tang et al., 2022). Self-determination theory further suggests that perceived social support satisfies an individual’s need for belonging, thereby enhancing intrinsic motivation to reciprocate and engage in responsible behaviors. Building on these theoretical frameworks, Hypothesis 2 is proposed: family communication patterns influence college students’ filial responsibility through the mediating role of perceived social support.

Gratitude plays a pivotal role in the formation and development of filial responsibility among college students. It is defined as the psychological tendency of individuals to recognize, emotionally respond to, and behave in ways that express appreciation for the benefits or assistance they receive. Gratitude fosters positive experiences and outcomes (McCullough et al., 2002). According to gratitude theory, filial responsibility in children arises from their recognition of the need to reciprocate the care and support provided by their parents (Keller, 2006). Huang (2023) further suggests that individuals with higher levels of gratitude tend to exhibit a stronger sense of responsibility. Ecological systems theory and family function theory emphasize the influence of parenting styles and the family environment in shaping the development of an individual’s gratitude (Peng and Dai, 2019; Long et al., 2014; Wang et al., 2020). According to the parent-child interaction model, open communication, the sharing of ideas, and the expression of concern between parents and children contribute to a supportive family environment. This, in turn, facilitates the expansion of external resources, improves the quality of family interactions, enhances interaction styles, and strengthens family resilience and adaptability, thereby fostering and shaping children’s gratitude traits (Fu, 2021). Based on these findings, Hypothesis 3 is proposed: family communication patterns influence college students’ filial responsibility through the mediating role of gratitude.

Attribution Theory suggests that individuals who make positive attributions regarding social support are more likely to acknowledge the contributions of those who have assisted them in the past, which in turn facilitates the development of gratitude-related cognitions and emotional experiences (Weiner, 1985). The Model of Person-Environment Transaction posits that as an individual’s perception of social support increases, gratitude emotions are more easily activated, which subsequently leads to the externalization of grateful behaviors (Ma et al., 2020). Building upon this framework, Hypothesis 4 is proposed: family communication patterns influence college students’ filial responsibility through chain-mediated effects involving perceived social support and gratitude.

## Materials and methods

### Participants

This study was conducted in November 2025 at a university in Fujian Province, employing a random sampling method. A total of 500 questionnaires were collected from an initial pool of 800 students. Since this study focuses on a specific group–university students–the selection of a single university as a sample for data collection is considered representative (Leedy and Ormrod, 2015; Salkind, 2017). Based on the established criteria for valid responses, 425 questionnaires were considered valid, resulting in an 85% validity rate. Further details are provided in Table 1. All methods were performed in accordance with the relevant guidelines and regulations. This study was approved by the Institutional Review Board (IRB) at Fujian Polytechnic Normal University of China (No. 2025-05) before data collection. Participants were informed of the study’s purpose, procedures, potential risks, and benefits. Informed consent was obtained from all participants prior to their inclusion in the study.

**TABLE 1:** Basic information.

Demographic variables	Category	Sample size (*N*)	Percentage (%) of the total sample
Sex	Male	108	25.41%
	Female	317	74.59%
Academic year	First year undergraduate	52	12.24%
	Second year undergraduate	82	19.29%
	Third year undergraduate	106	24.94%
	Fourth year undergraduate	97	22.82%
	Graduate students	88	20.71%
Type of children	Only child	135	31.76%
	Non-only child	290	68.24%

### Measures

#### Family communication patterns

The Family Communication Patterns Scale, as revised by Zhu (2021), was employed in this study. This scale was specifically designed to assess family communication patterns among college students and was tailored to the Chinese cultural context. The internal consistency coefficient (Cronbach’s α) of the scale was 0.840, indicating adequate reliability. In the present study, the internal consistency for the Conversation Orientation of the Family Communication Patterns Scale was 0.937. Confirmatory factor analysis (CFA) results indicated χ^2^ = 399.106, *df* = 77, *CFI* = 0.915, *TLI* = 0.899, *RMSEA* = 0.100, and *SRMR* = 0.046, demonstrating strong structural validity. Factor loadings for each item in this orientation ranged from 0.607 to 0.861, further supporting the aggregation of a total score for the Conversation Orientation of family communication patterns. Similarly, the internal consistency coefficient for the Conformity Orientation was 0.918, indicating high reliability. CFA for this dimension yielded χ^2^ = 196.184, *df* = 35, *CFI* = 0.936, *TLI* = 0.918, *RMSEA* = 0.104, and *SRMR* = 0.041, further demonstrating robust structural validity. Factor loadings for each item in this orientation ranged from 0.629 to 0.821, enabling the aggregation of a total score for the Conformity Orientation of family communication patterns.

#### Filial responsibility

The Filial Responsibility Scale was adapted from Du’s (2010) Filial Responsibility Behavior Scale, which was designed to assess the degree to which college students fulfill their filial responsibilities. Higher scores on the scale reflect a greater degree of fulfillment of filial responsibilities. The internal consistency coefficient (α) of the scale was 0.910 in the original study, and 0.924 in the current study. As the original scale comprises five dimensions, this study employed item parceling. Confirmatory factor analysis (CFA) revealed satisfactory model fit indices: χ^2^ = 49.135, *df* = 5, *CFI* = 0.961, *TLI* = 0.922, *RMSEA* = 0.145, *SRMR* = 0.036, indicating that the scale demonstrates good structural validity. Factor loadings for the five dimensions ranged from 0.653 to 0.871, which supports the aggregation of these dimensions into a single composite score representing filial responsibility.

#### Perceived social support

The Perceived Social Support Scale was adapted from the revised version by Yan and Xue (2006), specifically designed for college student populations. Higher total scores on the scale reflect a higher level of perceived social support. The internal consistency coefficient (α) of the scale was 0.930, and in the current study, the α coefficient was 0.905. Confirmatory factor analysis (CFA) yielded satisfactory fit indices: χ^2^ = 195.052, *df* = 27, *CFI* = 0.922, *TLI* = 0.896, *RMSEA* = 0.121, *SRMR* = 0.044, thereby confirming that the scale demonstrates good structural validity. Factor loadings for individual items ranged from 0.663 to 0.816, suggesting that the items can be reliably aggregated into a single composite score representing perceived social support.

#### Gratitude

The Gratitude Scale used in this study is the Chinese version (CGQ–6), revised by Wei et al. (2011) based on the original scale developed by McCullough et al. (2002). This version has been validated, exhibiting satisfactory reliability (α = 0.796), in line with the established standards for psychological research. In the current study, the internal consistency coefficient (α) was 0.899. Confirmatory factor analysis (CFA) revealed robust model fit indices: χ^2^ = 54.489, *df* = 9, *CFI* = 0.971, *TLI* = 0.952, *RMSEA* = 0.109, *SRMR* = 0.033, indicating strong structural validity of the scale. Factor loadings for each item ranged from 0.630 to 0.912, supporting the aggregation of these items into a single composite score for gratitude.

### Statistical analysis

Initially, exploratory factor analysis (EFA) was performed using SPSS 26.0 to examine the items corresponding to four key variables: family communication patterns, filial responsibility, perceived social support, and gratitude. This analysis was intended to assess potential common method bias in the current study. Subsequently, Pearson’s correlation analysis were computed to examine the interrelationships among the four variables. Finally, structural equation modeling (SEM) was implemented using Mplus 8.3 to assess the chain mediating effects of perceived social support and gratitude between family communication patterns and filial responsibility.

## Results

### Analysis of common method bias

A common method bias test was performed in this study according to the methodology proposed by Zhou and Long (2004). The results revealed the existence of 11 common factors with eigenvalues greater than 1, collectively accounting for 64.780% of the total variance. Specifically, the first factor accounted for 31.092% of the variance, which remains below the 40% threshold. Therefore, no substantial common method bias was detected in this study.

### Correlation analysis

This study performed a correlation analysis of four key variables, with the findings summarized in Table 2. The family communication pattern-conversation orientation was significantly positively associated with filial responsibility (*r* = 0.663, *p* < 0.01), perceived social support (*r* = 0.521, *p* < 0.01), and gratitude (*r* = 0.323, *p* < 0.01). In contrast, the family communication pattern-conformity orientation showed significant negative associations with filial responsibility (*r* = −0.428, *p* < 0.01), perceived social support (*r* = −0.381, *p* < 0.01), and gratitude (*r* = −0.263, *p* < 0.01). Furthermore, filial responsibility was significantly positively associated with perceived social support (*r* = 0.558, *p* < 0.01) and gratitude (*r* = 0.432, *p* < 0.01), while perceived social support was also significantly positively associated with gratitude (*r* = 0.557, *p* < 0.01).

**TABLE 2:** The interrelationships among the various variables.

Variables	1	2	3	4	5
1 Conversation orientation	1	1	1	1	1
2 Conformity orientation	−0.573^**^				
3 Filial responsibility	0.663^**^	−0.428^**^			
4 Perceived social support	0.521^**^	−0.381^**^	0.558^**^		
5 Gratitude	0.323^**^	−0.263^**^	0.432^**^	0.557^**^	

^**^p < 0.01.

### Examination of the chain-mediated effect

Considering the negligible influence of demographic variables on the four primary variables, they were excluded from control in this analysis. A structural equation model (SEM) was specified using Mplus 8.3 to assess the mediation effects. The independent variable was family communication patterns, the dependent variable was filial responsibility, and perceived social support and gratitude acted as mediators. The significance of the mediation effects was assessed using the bootstrap method, where the 95% confidence interval was used to determine whether it contained zero. If the 95% confidence interval did not contain zero, the mediation effect was considered significant; conversely, if the interval contained zero, the effect was deemed non-significant (Taylor et al., 2008).

**FIGURE 1:**
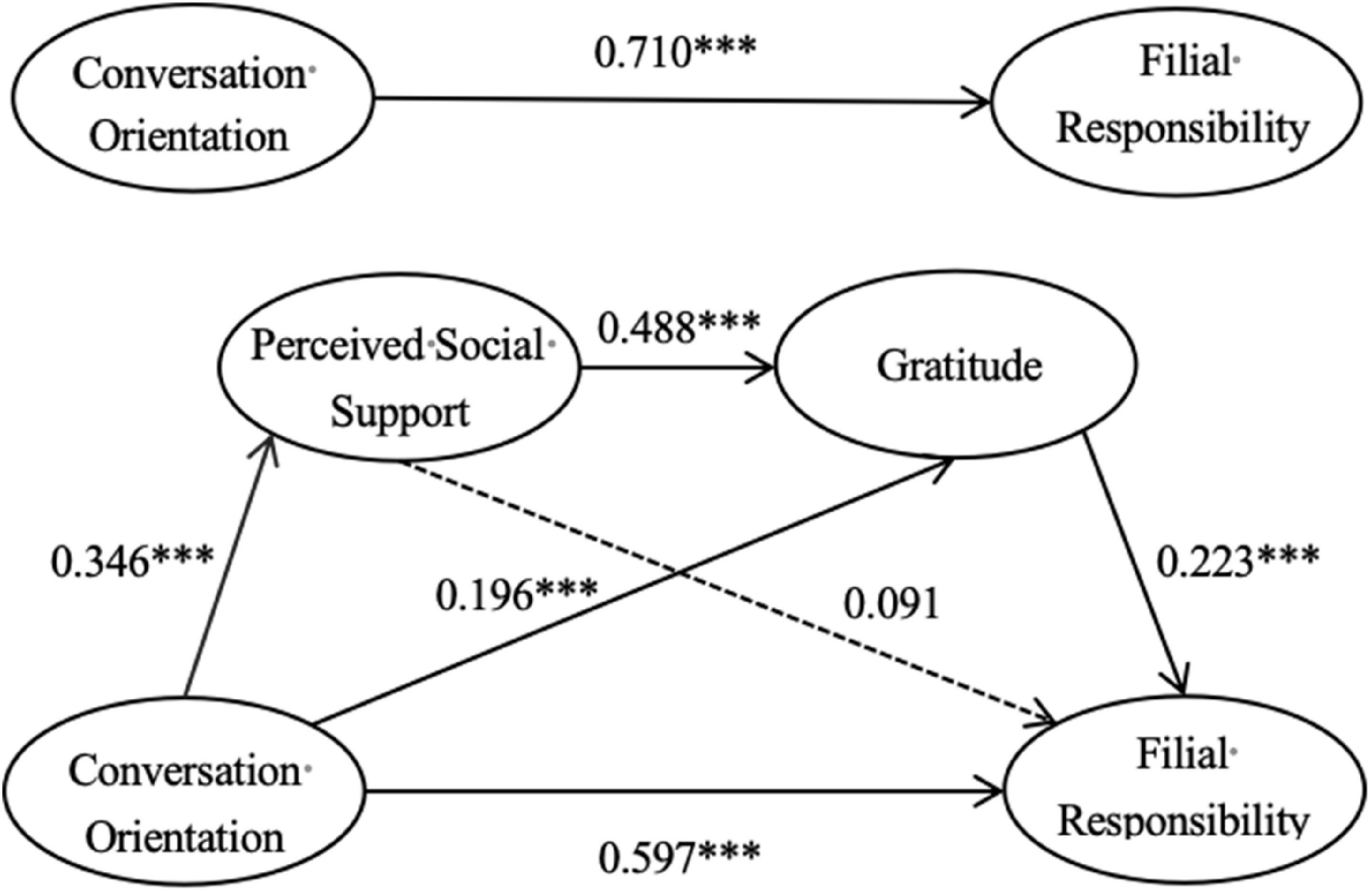
Path diagram illustrating the mediating effects of perceived social support and gratitude in the relationship between family communication patterns (conversation orientation) and filial responsibility. ***p < 0.001.

In the present study, we developed a mediation model with family communication patterns, specifically the conversation orientation, as the independent variable, filial responsibility as the dependent variable, and perceived social support and gratitude as mediators. The model fit indices are as follows: χ^2^ = 1181.763, *df* = 489, χ^2^/*df* = 2.42 (<3), *p* < 0.001, *CFI* = 0.924, *TLI* = 0.918, *RMSEA* = 0.058, and *SRMR* = 0.050, indicating an adequate model fit. The results of the analysis support the validity of the mediation model. Path analysis indicated that the family communication pattern–specifically the conversation orientation–directly predicted filial responsibility (β = 0.710, *p* < 0.001). When perceived social support and gratitude were included as mediators, the conversation orientation of family communication patterns continued to significantly predict filial responsibility (β = 0.597, *p* < 0.001), perceived social support (β = 0.346, *p* < 0.001), and gratitude (β = 0.196, *p* < 0.001). Perceived social support significantly predicted gratitude (β = 0.488, *p* < 0.001), but did not significantly predict filial responsibility (β = 0.091, *p* > 0.05). Gratitude, however, was found to significantly predict filial responsibility (β = 0.223, *p* < 0.001). For further details, please refer to Figure 1 and Table 3.

**TABLE 3:** The chain mediating effects in the model.

Predictor variables	Outcome variables	β	*p*	*Ses*
Conversation orientation	Filial responsibility	0.710***	0.000	0.032
Conversation orientation	Filial responsibility	0.597***	0.000	0.042
	Perceived social support	0.346***	0.000	0.053
	Gratitude	0.196***	0.000	0.055
Perceived social support	Filial responsibility	0.091	0.085	0.053
Perceived social support	Gratitude	0.488***	0.000	0.058
Gratitude	Filial responsibility	0.223***	0.000	0.056

^***^p < 0.001.

The significance of the mediation effects was evaluated using a non-parametric percentile bootstrap method with 5000 resamples, as presented in Table 4. The results indicated that the total effect of family communication patterns (conversation orientation) on college students’ filial responsibility was 0.710 (LLCI = 0.645, ULCI = 0.769), with a direct effect of 0.597 (LLCI = 0.510, ULCI = 0.677) and a total indirect effect of 0.113 (LLCI = 0.068, ULCI = 0.167). The 95% confidence intervals for all effects excluded zero, and the *p*-value was less than 0.001, confirming statistical significance. Specifically, the mediation effect of family communication patterns (conversation orientation) → gratitude → filial responsibility was 0.044 (LLCI = 0.016, ULCI = 0.084), and the mediation effect of family communication patterns (conversation orientation) → perceived social support → gratitude → filial responsibility was 0.038 (LLCI = 0.019, ULCI = 0.066). Both effects were statistically significant, as evidenced by 95% confidence intervals excluding zero. However, the mediation effect of family communication patterns (conversation orientation) → perceived social support → filial responsibility was 0.031 (LLCI = −0.003, ULCI = 0.074), with a confidence interval that included zero. This suggests that family communication patterns (conversation orientation) do not indirectly predict filial responsibility through perceived social support. Consequently, while family communication patterns (conversation orientation) influence filial responsibility through gratitude and through a sequential mediation involving perceived social support and gratitude, they do not exert an indirect effect through perceived social support alone.

**TABLE 4:** Bootstrap estimations of the path coefficients in the model.

Prediction path	Effect value	*SE*	95% confidence interval	Effect size
Total effect	0.710	0.032	[0.645, 0.769]	
Direct effect	0.597	0.042	[0.510, 0.677]	84.1%
Total indirect effect	0.113	0.025	[0.068, 0.167]	15.9%
Indirect effect 1	0.031	0.019	[−0.003, 0.074]	4.4%
Indirect effect 2	0.044	0.017	[0.016, 0.084]	6.2%
Indirect effect 3	0.038	0.012	[0.019, 0.066]	5.4%

Effect size = mediated effect/total effect. Indirect effect 1: family communication patterns (conversation orientation) → perceived social support → filial responsibility. Indirect effect 2: family communication patterns (conversation orientation) → gratitude → filial responsibility. Indirect effect 3: family communication patterns (conversation orientation) → perceived social support → gratitude → filial responsibility.

**FIGURE 2:**
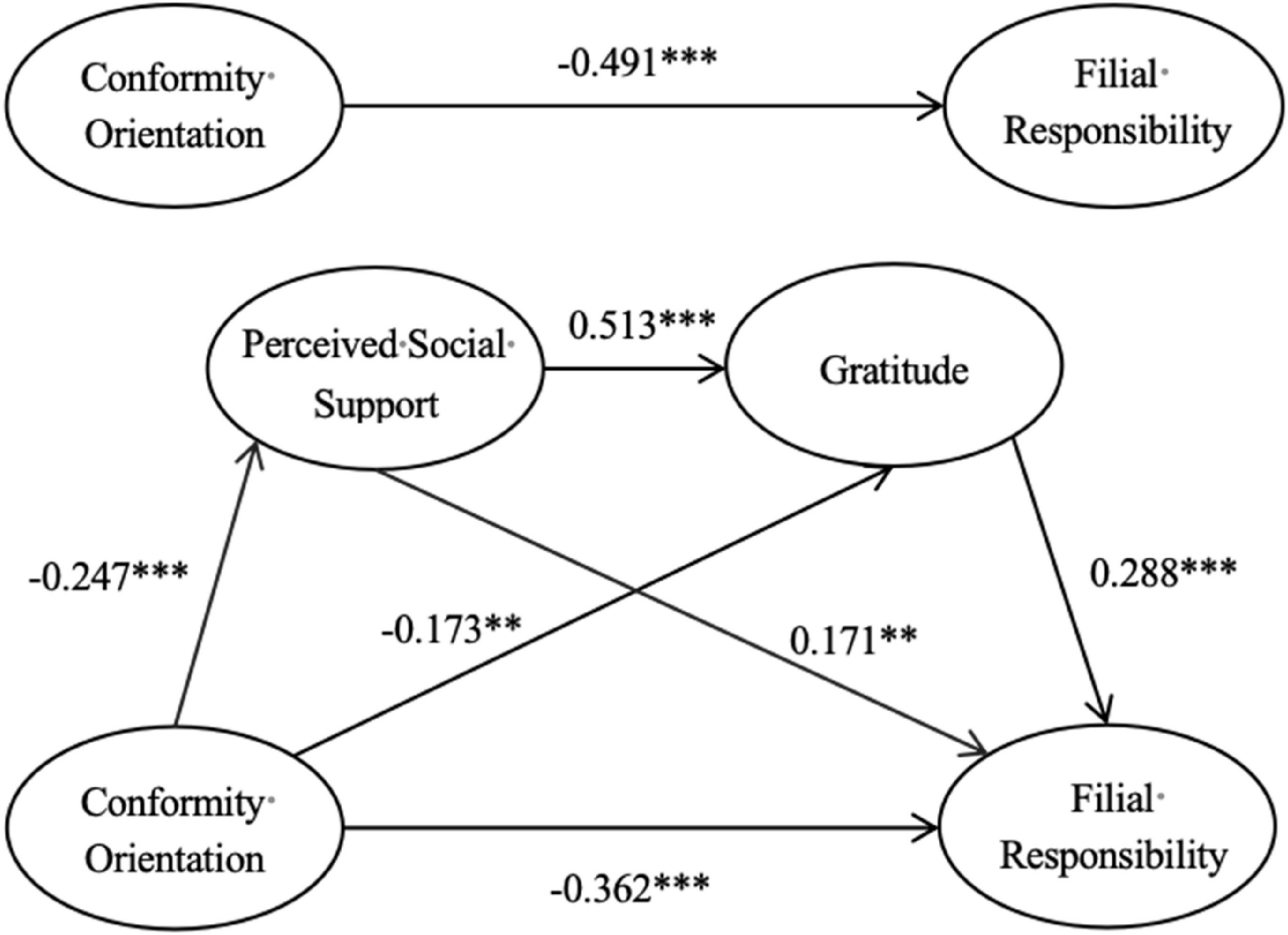
Path diagram illustrating the mediating effects of perceived social support and gratitude in the relationship between family communication patterns (conformity orientation) and filial responsibility. **p < 0.01, ***p < 0.001.

A mediational model was constructed with family communication patterns, specifically the conformity orientation, as the independent variable, filial responsibility as the dependent variable, and perceived social support and gratitude as mediating variables. The model fit indices are as follows: χ^2^ = 909.933, *df* = 371, χ^2^/*df* = 2.45 (<3), *p* < 0.001, *CFI* = 0.930, *TLI* = 0.924, *RMSEA* = 0.059, *SRMR* = 0.047, indicating a satisfactory fit. This analysis supports the validity of the mediational model. The path analysis reveals that the family communication pattern–specifically the conformity orientation–directly and negatively predicts filial responsibility (β = −0.491, *p* < 0.001). After incorporating perceived social support and gratitude as mediators, the conformity orientation of family communication patterns significantly predicts filial responsibility (β = −0.362, *p* < 0.001), perceived social support (β = −0.247, *p* < 0.001), and gratitude (β = −0.173, *p* < 0.01). Furthermore, perceived social support significantly predicts gratitude (β = 0.513, *p* < 0.001) and filial responsibility (β = 0.171, *p* < 0.01), while gratitude significantly predicts filial responsibility (β = 0.288, *p* < 0.001). For further details, please refer to Figure 2 and Table 5.

**TABLE 5:** The chain mediating effects in the model.

Predictor variables	Outcome variables	β	*p*	*Ses*
Conformity orientation	Filial responsibility	−0.491^***^	0.000	0.046
Conformity orientation	Filial responsibility	−0.362^***^	0.000	0.053
	Perceived social support	−0.247^***^	0.000	0.054
	Gratitude	−0.173^**^	0.001	0.050
Perceived social support	Filial responsibility	0.171^**^	0.004	0.059
Perceived social support	Gratitude	0.513^***^	0.000	0.052
Gratitude	Filial responsibility	0.288^***^	0.000	0.060

^**^p < 0.01,

^***^p < 0.001.

The significance of the mediation effects was evaluated using a non-parametric percentile bootstrap method with 5000 resamples, as presented in Table 6. The results indicate that the total effect of family communication patterns (conformity orientation) on university students’ filial responsibility was −0.491 (LLCI = −0.572, ULCI = −0.388), with a direct effect of −0.362 (LLCI = −0.462, ULCI = −0.252) and an indirect effect of −0.129 (LLCI = −0.190, ULCI = −0.080). The 95% confidence intervals for all effects excluded zero, and the *p*-value was less than 0.001. Specifically, the mediation effects of family communication patterns (conformity orientation) → perceived social support → filial responsibility was −0.042 (LLCI = −0.088, ULCI = −0.012); family communication patterns (conformity orientation) → gratitude → filial responsibility was −0.023 (LLCI = −0.091, ULCI = −0.017); and the chain mediation of family communication patterns (conformity orientation) → perceived social support → gratitude → filial responsibility was −0.037 (LLCI = −0.063, ULCI = −0.018). All three mediation pathways were statistically significant. These findings suggest that family communication patterns (conformity orientation) can influence students’ filial responsibility both through the mediating roles of perceived social support and gratitude, as well as through the chain mediation involving both perceived social support and gratitude.

**TABLE 6:** Bootstrap estimations of the path coefficients in the model.

Prediction path	Effect value	*SE*	95% confidence interval	Effect size
Total effect	−0.491	0.046	[−0.572, −0.388]	
Direct effect	−0.362	0.053	[−0.462, −0.252]	73.7%
Total indirect effect	−0.129	0.028	[−0.190, −0.080]	26.3%
Indirect effect 1	−0.042	0.018	[−0.088, −0.012]	8.6%
Indirect effect 2	−0.023	0.019	[−0.091, −0.017]	4.7%
Indirect effect 3	−0.037	0.011	[−0.063, −0.018]	7.5%

Effect size = mediated effect/total effect. Indirect effect 1: family communication patterns (conformity orientation) → perceived social support → filial responsibility. Indirect effect 2: family communication patterns (conformity orientation) → gratitude → filial responsibility. Indirect effect 3: family communication patterns (conformity orientation) → perceived social support → gratitude → filial responsibility.

## Discussion

### The influence of family communication patterns on filial responsibility among college students

The study reveals a significant positive correlation between the conversation orientation of family communication patterns and filial responsibility, whereas the conformity orientation is negatively correlated with filial responsibility. These findings are consistent with previous research. Scruggs and Schrodt (2021) assert that a higher conversation orientation in family communication patterns promotes open and enjoyable exchanges, thereby enhancing children’s satisfaction with the family and fostering closer relationships with parents. In contrast, the conformity orientation does not yield similar predictive outcomes. Schrodt (2009) highlights that children from families with a strong conversation orientation are more likely to perceive familial advantages and report greater family satisfaction, while those from families with a lower conversation orientation experience fewer such benefits. Koerner et al. (2017) found that in conformity-oriented families, parents emphasize the importance of authority and conformity, often making decisions unilaterally and excluding children from the decision-making process. This approach tends to diminish children’s satisfaction and sense of belonging within the family (Horstman et al., 2018). Previous studies have indicated that children who establish stronger emotional bonds and higher relationship quality with their parents are more likely to exhibit greater filial responsibility (Gans et al., 2009; Kim et al., 2014; Hwang et al., 2018; Swartz, 2009; Lin et al., 2023). Liu et al. (2018) argue that children perceive filial responsibility primarily due to the positive emotional experiences they have during communication with their parents.

### The mediating effect of perceived social support in the relationship between family communication patterns and filial responsibility in college students

The study demonstrates that perceived social support does not act as a partial mediator in the relationship between the conversation orientation of family communication patterns and filial responsibility. This finding may be attributed to the small effect size between perceived social support and filial responsibility (Wen et al., 2016). According to Wassertheil and Cohen (1970), an effect size of *d* = 0.2 indicates a small effect, and in the present study, the effect size between perceived social support and filial responsibility was *d* = 0.091 (see Figure 2), which also falls within the small effect range. As noted by Wen et al. (2016), small effect sizes are typically regarded as lacking practical significance unless they exert a substantial impact. Therefore, from this perspective, the relationship between perceived social support and filial responsibility may lack practical significance, despite the divergence from prior research (Wang, 2023; Yang, 2023). However, when perceived social support and gratitude were sequentially incorporated into a mediation model, the effect of the conversation orientation of family communication patterns on filial responsibility became significant. This suggests that gratitude plays a more pivotal role in mediating the relationship between the conversation orientation of family communication and filial responsibility. Specifically, it appears that college students are only able to perceive filial responsibility after recognizing social support and experiencing an increase in their level of gratitude. This may be due to the stronger influence of gratitude, as compared to perceived social support, on filial responsibility. This finding aligns with the results of Huang (2023), who found that higher levels of gratitude are associated with a stronger sense of responsibility. Moreover, the significant positive correlation between the conversation orientation of family communication and perceived social support is consistent with previous studies (Bevan et al., 2019; Bosch et al., 2012; Xu et al., 2016; Yang et al., 2022). This indicates that in democratic and open family environments, communication and the willingness to express oneself freely facilitate the acquisition of both familial and social support.

The study demonstrates that perceived social support partially mediates the relationship between the conformity orientation of family communication patterns and filial responsibility. The negative correlation observed between the conformity orientation of family communication patterns and perceived social support is consistent with previous research (Bevan et al., 2019; Bosch et al., 2012; Xu et al., 2016; Yang et al., 2022). This finding may be attributed to the ineffective or obstructed communication inherent in the conformity orientation, which limits children’s perceptions of parental care, understanding, and support. Consequently, this hampers their self-disclosure and interpersonal relationship maintenance, and may also constrain the support they receive from peers and teachers. The positive correlation between perceived social support and filial responsibility is consistent with previous studies, which have suggested that filial responsibility is maintained through social support (Zhang et al., 2018). Greater family support has been shown to foster a more positive attitude toward filial responsibility among children (Ron, 2019; Wang, 2023; Yang, 2023). In comparison to the conversation orientation of family communication patterns, perceived social support plays a more significant mediating role between the conformity orientation and filial responsibility. This could be explained by the relatively lower levels of gratitude among children exposed to the conformity orientation, which diminishes its impact on filial responsibility, thus enhancing the mediating effect of perceived social support.

### The mediating effect of gratitude in the relationship between family communication patterns and filial responsibility in college students

This study reveals that gratitude serves as a partial mediator in the relationship between family communication patterns–specifically the orientations of conversation and conformity–and filial responsibility. The conversation orientation of family communication patterns is significantly and positively correlated with gratitude, while the conformity orientation exhibits a negative correlation. This finding is consistent with previous research (Fu, 2021; Leng, 2022). According to the parent-child interaction model, in a high conversation orientation, open communication, the exchange of thoughts and ideas, and the expression of concern between parents and children foster a warm and supportive family environment. This environment helps children expand their external support networks, improves the quality and nature of family interactions, and enhances their social adaptability, thereby promoting the development of gratitude (Fu, 2021). In contrast, children raised in a high conformity orientation tend to experience lower levels of satisfaction and emotional closeness with family members (Horstman et al., 2018; Scruggs and Schrodt, 2021), which impedes the development of gratitude. Both ecological systems theory and family function theory underscore the synergistic effects of parenting styles and family environments on the emergence and development of gratitude in individuals (Peng and Dai, 2019).

A significant positive correlation exists between gratitude and filial responsibility, a finding that aligns with prior research. Keller (2006) proposed that filial responsibility can be understood through the gratitude theory, suggesting that children fulfill their filial responsibility out of a profound sense of gratitude for the nurturing and care provided by their parents. Similarly, Huang (2023) emphasized that higher levels of gratitude are linked to a greater sense of responsibility. Furthermore, research by Fu (2021) has demonstrated that open, egalitarian communication within the family fosters children’s gratitude, which, in turn, strengthens their recognition of and commitment to fulfilling filial responsibilities.

### The chain mediation of perceived social support and gratitude in the link between family communication patterns and filial responsibility in college students

This study demonstrates that perceived social support and gratitude act as chain mediators between both the conversation and conformity orientations of family communication patterns and filial responsibility. Family communication patterns arise from interactions within the family, which both reflect and influence the way family members engage with each other. These patterns play a crucial role in shaping family interactions, relationships, and dynamics, which significantly influence children’s cognitive, emotional, and behavioral development. Attachment theory, as proposed by Mikulincer and Shaver (2007) and Waters and Waters (2006), introduces the concept of the “secure base schema,” which suggests that an individual’s interactions with attachment figures are encoded as memory-based schemas, influencing cognitive, emotional, and behavioral outcomes. This theoretical framework provides insight into the mediating role of perceived social support and gratitude between the conversation and conformity orientations of family communication patterns and filial responsibility. In families characterized by a high conversation orientation, active dialogue and positive interactions between parents and children promote the development of children’s self-awareness and cognitive capacities. This facilitates children’s recognition of the benevolence and support provided by significant others, thereby fostering gratitude toward their parents. Such gratitude, in turn, cultivates filial responsibility, which manifests in behaviors directed at caring for and supporting parents. Conversely, in families with a high conformity orientation, parent–child interactions tend to be more distant and rigid. Consequently, children are less likely to perceive the support of significant others, thereby impeding the development of both gratitude and filial responsibility toward their parents.

Social Cognitive Theory offers a framework to understand the chain mediating role of perceived social support and gratitude in the relationship between both the conversation and conformity orientations of family communication patterns and filial responsibility. According to this theory, there is an interaction among environmental factors, individual cognitive factors, and behavior. Specifically, environmental factors, such as family communication patterns, influence individual cognitive factors (i.e., perceived social support and gratitude), while individual cognitive factors, particularly gratitude, subsequently shape individual behaviors, including filial responsibility. Perceived social support refers to an individual’s awareness and interpretation of long-standing social support in their life (Xiao, 1994), characterized by the subjective perception and evaluation of the degree of external support (Zimet et al., 1988). Gratitude is an emotional disposition that arises when an individual recognizes the assistance provided by others, leading to feelings of appreciation and a willingness to reciprocate (Wang and Du, 2019). According to the main effects model of social support (Malecki and Demaray, 2002), positive, warm, and egalitarian communication interactions foster children’s awareness of their parents’ care, love, understanding, and support. This type of interaction enables individuals to perceive higher levels of social support, which, in turn, promotes greater gratitude in their daily lives. Moreover, Self-Determination Theory posits that when individuals recognize and internalize external support, they experience the satisfaction of both emotional and psychological needs, thereby interpreting perceived support as a benevolent act from others, which triggers feelings of gratitude. Finally, the emergence of gratitude in children encourages the development of filial responsibility toward their parents (Keller, 2006; Huang, 2023). This framework also explains that in family environments with clearly defined hierarchical structures and prioritized family interests, children may struggle to perceive their parents’ affection, warmth, and support. This, in turn, may hinder the development of gratitude and, consequently, their sense of filial responsibility.

## Limitations and future directions

This study employs a cross-sectional design, which limits the ability to draw causal inferences about the relationships between variables. Consequently, future studies should adopt longitudinal designs to obtain a more in-depth understanding of the developmental trajectories of these variables and their causal relationships.

Due to limitations in data collection stemming from logistical constraints and practical considerations, this study was confined to students from a single university in Fujian Province, which may limit the representativeness of the sample. Therefore, future research should seek to broaden the geographic scope of the sample by incorporating additional regions and diverse populations, thereby improving the generalizability and external validity of the findings.

The measurement tool used in this study solely focused on college students’ perceptions of family communication patterns, which may lead to bias by overlooking the perspectives and experiences of other family members. Therefore, future studies should incorporate parents as participants to obtain data from diverse perspectives, thus facilitating a more objective and comprehensive understanding of family communication patterns within Chinese households.

The data collection method employed in this study was limited to survey questionnaires, which may have undermined the objectivity of the measurement, potentially introducing bias. Therefore, future studies should consider integrating experimental and interview methodologies to improve the robustness and validity of the collected data.

## Practical implications

(1) Family Aspect: Creating a Safe and Warm Communication Environment

This study indicates that the conversation orientation of family communication patterns is significantly positively correlated with other variables, while the conformity orientation shows a significant negative correlation with other variables. Therefore, to foster children’s understanding of social support, gratitude, and to enhance their sense of filial responsibility, family communication patterns should be optimized in the following ways.

First, families should encourage children to freely express their opinions and needs, thereby creating a safe and warm communicative environment. Children growing up in families with a high conversation orientation typically report higher satisfaction with their families and closer relationships with their parents. This, in turn, facilitates the children’s recognition and practice of their filial responsibilities.

Second, children’s individual rights should be valued and respected, and parental authority should not be the sole means of exerting control. As independent individuals, children should have the right to express their views and receive respect from their parents, rather than being forced into obedience through coercive means. Otherwise, such coercion can lead to negative emotions in children, distancing them from their parents and reducing their willingness to care for and respect them.

(2) School Aspect: Emphasizing the Cultivation and Education of Social Support and Gratitude

This study demonstrates that perceived social support and gratitude can enhance college students’ filial responsibility. Therefore, first, schools and educators should provide sufficient care and support in students’ academic and interpersonal interactions, helping them recognize social support from teachers, family, and peers, while also fostering a sense of gratitude. Second, opportunities should be provided for students to express and practice gratitude, guiding them to experience the positive personal impact of expressing gratitude, thereby further enhancing their willingness to express gratitude, which lays a foundation for assuming filial responsibility.

(3) Social Aspect: Focusing on Changes in Family Communication Patterns and Providing Guidance

With the social and cultural changes, along with the spread of Western liberal and egalitarian ideologies, the concept of filial responsibility and family communication patterns are undergoing significant transformations. Firstly, community service centers should develop courses aimed at promoting harmonious family communication, and organize joint learning sessions for parents and children. The goal is to raise awareness of the significance of family communication patterns on children’s development, ultimately aiding in the establishment of appropriate communication methods. Secondly, community service centers should regularly organize parent-child activities related to filial responsibility, helping children gradually understand the concept of filial responsibility while also emphasizing the importance for parents to provide opportunities for children to practice these responsibilities. Finally, community service centers may organize regular participation in community service for children, allowing them to experience the care and support among community members. This process can foster a sense of gratitude and awareness of social support.

## Conclusion

The conversation orientation of family communication patterns significantly and positively predicts college students’ filial responsibility, whereas the conformity orientation significantly and negatively predicts it. Perceived social support partially mediates the relationship between the conformity orientation of family communication patterns and students’ filial responsibility. Gratitude also partially mediates the relationship between both the conversation and conformity orientations of family communication patterns and filial responsibility. Moreover, perceived social support and gratitude together exert a chain-mediating effect between the conversation and conformity orientations of family communication patterns and college students’ filial responsibility.

## Data Availability

The raw data supporting the conclusions of this article will be made available by the authors, without undue reservation.
